# An Infinitesimal Model for Quantitative Trait Genomic Value Prediction

**DOI:** 10.1371/journal.pone.0041336

**Published:** 2012-07-18

**Authors:** Zhiqiu Hu, Zhiquan Wang, Shizhong Xu

**Affiliations:** 1 Department of Botany and Plant Sciences, University of California Reverside, Reverside, California, United States of America; 2 Department of Agricultural Food and Nutritional Science, University of Alberta, Edmonton, Alberta, Canada; University of California, Irvine, United States of America

## Abstract

We developed a marker based infinitesimal model for quantitative trait analysis. In contrast to the classical infinitesimal model, we now have new information about the segregation of every individual locus of the entire genome. Under this new model, we propose that the genetic effect of an individual locus is a function of the genome location (a continuous quantity). The overall genetic value of an individual is the weighted integral of the genetic effect function along the genome. Numerical integration is performed to find the integral, which requires partitioning the entire genome into a finite number of bins. Each bin may contain many markers. The integral is approximated by the weighted sum of all the bin effects. We now turn the problem of marker analysis into bin analysis so that the model dimension has decreased from a virtual infinity to a finite number of bins. This new approach can efficiently handle virtually unlimited number of markers without marker selection. The marker based infinitesimal model requires high linkage disequilibrium of all markers within a bin. For populations with low or no linkage disequilibrium, we develop an adaptive infinitesimal model. Both the original and the adaptive models are tested using simulated data as well as beef cattle data. The simulated data analysis shows that there is always an optimal number of bins at which the predictability of the bin model is much greater than the original marker analysis. Result of the beef cattle data analysis indicates that the bin model can increase the predictability from 10% (multiple marker analysis) to 33% (multiple bin analysis). The marker based infinitesimal model paves a way towards the solution of genetic mapping and genomic selection using the whole genome sequence data.

## Introduction

The infinitesimal model of quantitative traits has dominated quantitative genetics for over 70 years until the end of 1980’s when interval mapping of quantitative trait loci (QTL) was first introduced by Lander and Botstein [1]. The infinitesimal model states that a quantitative trait is controlled by an infinite number of loci and each locus has an infinitely small effect [2], [3], the model is also called the polygenic model [4]. Under this model, the effect of each locus is unrecognizable and thus these loci must be studied collectively under the general framework of classical quantitative genetics [5]. Consider that all the small effect genes are linearly arranged in a genome, the infinitesimal model is essentially a continuous genome model. Prior to the genome era, it was impossible to directly validate the infinitesimal model other than using resemblance between relatives to estimate the collective contribution of all loci on the genome for a quantitative trait. With the advent of advanced molecular technology, DNA marker data are available and they have been used to identify major genes for some quantitative traits [6], [7], [8], [9]. The major gene identification approach is based on a modified version of the infinitesimal model, called oligogenic model [10], which states that a quantitative trait is controlled by a few genes with large effects and many genes with small effects. In the context of linkage analysis, these genes are called QTL. Using markers to detect QTL is called QTL mapping. QTL mapping may only detect genes with large and median sized effects. The small effect QTL may not be detected separately at all for the sample sizes affordable in a current experiment.

The high density SNP data provide a way to capture the polygene. Using a Bayesian approach, effects of the high density markers can be estimated jointly to predict the genomic values for a quantitative trait without performing marker selection. This approach is called genomic selection [11], [12]. Simulation studies showed that genomic selection using markers alone can fit the model with an accuracy up to 85% [11]. The 85% accuracy is the correlation between the true genetic values and the predicted values of individuals in the next generation. True genetic values are only known in simulation studies. In real data analysis, the predictability of a model must be drawn from a cross validation study. The predictability obtained from cross validation and the goodness of model fit do not necessarily agree to each other. Starting from a small number of markers, they may both increase as the number of markers increases. Further increasing the number of markers may continue to increase the goodness of model fit but the predictability may fall down [13], [14].

In the genome era, the number of SNP markers can easily reach up to one million [15]. In the near future, one million SNP markers may be available for many species. No methods are available to estimate one million effects jointly in a single model. If we treat each marker as a QTL, the model is virtually an infinitesimal model. How to handle such a model with an infinite dimension remains a challenging problem. We will introduce a continuous genome model by replacing the summation of infinite terms by an integral and then used a numerical integration approach to calculate the integral. The numerical integration will be achieved via dividing the entire genome into many small intervals (also called bins). The bin effects are then subject to estimation, instead of the individual marker effects. Each bin may contain many markers and the bin effect represents the total effects of all markers within that bin. This special model dimension reduction approach has never been proposed before for genomic prediction.

We first present an infinitesimal model to handle populations initiated from line crossing experiments. Such populations represent the ones with high linkage disequilibrium. We then extend to model to handle populations with low or no linkage disequilibrium.

## Materials and Methods

### Concept of the Infinitesimal Model

Let 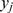 be the observed phenotypic value for individual *j* in a population of size *n*. The linear model for a usual regression analysis is
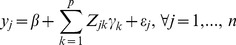
(1)where 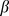 is the intercept, 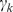 is the effect of locus *k*, 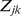 is a known genotypic indicator variable for individual *j* at locus *k* and 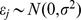 is the residual error with an unknown variance 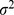. The genotype indicator variable 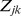 for locus *k* is defined as
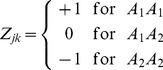
(2)where 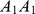, 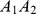 and 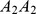 are the three genotypes for locus k. Note that p is the number of loci included in the model. When 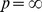, the model becomes



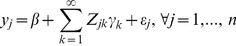
(3)This is the infinitesimal model of quantitative trait [2]. The regression coefficient 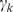 cannot be estimated because (a) the model has an infinite size and (b) the model is ill-conditioned, e.g., high multicollinearity. Now let us replace *k* by the corresponding genome location of the locus, denoted by 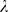, which is continuous and ranges from 0 to 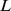 where 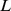 is the genome size. The infinitesimal model may be replaced by
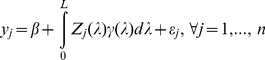
(4)where 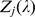 is known for genome saturated with markers and 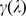 is the genetic effect expressed as an unknown function of the genome location. Our purpose is to estimate 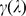 using the data, which include 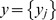 and 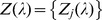. The parameters include 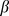, 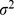 and 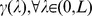. The estimation should be obtained by optimizing some well-defined criteria, such as minimizing the sum of squared differences
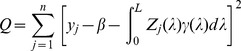
(5)or maximizing the log likelihood function



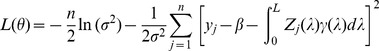
(6)The likelihood function is obtained based on the assumed normal distribution 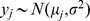, where 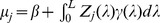 is the expectation of 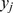. The ultimate purpose of the infinitesimal model is to find the unknown function 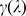 so that we can use
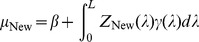
(7)to predict an unobserved 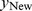, where the subscript New means a new individual with known 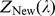 but unknown 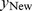.

The infinitesimal model given in equation (3) is represented by the continuous genome model in equation (4). There is no explicit expression of the integral and thus numerical integration is required to approximate the integral. Let us divide the entire genome by *m* bins (a bin is also called an interval of the genome) indexed by *k* for the *k* ^th^ bin. Let 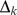 be the size of bin *k*, which may be the same for all bins or vary across different bins. The numerical approximation of the continuous genome model is
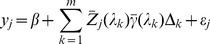
(8)where 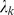 is the middle point position of the *k* ^th^ bin in the genome, 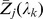 is the average value of 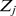 for all markers covered by the *k* ^th^ bin, 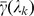 is the average effect of all QTL in that bin and 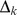 is the size of this bin. Note that 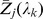 is a known quantity and 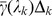 is unknown that is subject to estimation. When 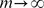, the bin size 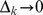 and the model becomes the exact continuous genome model. The unknown function 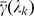 can be estimated from the data. The number of bins *m* depends on the sample size *n* and the level of linkage disequilibrium. A larger sample size allows a larger *m*. High linkage disequilibrium can be dealt with a small number of bins. Because 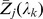 is an average value of all markers within the bin, markers of the entire genome have been utilized. When the interval 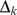 covers a large number of markers, 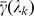 can be very small (not estimable). This is why the model is called the infinitesimal model.

In reality, the number of markers within a bin can be finite. Let 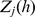 and 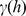 be the genotype indicator variable and the effect of marker *h* in bin *k* for 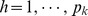 where 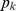 is the number of markers in bin *k* for 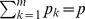. In real data analysis, the bin size 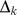 is replaced by the number of markers in bin *k* and the genome size is represented by the total number of markers *p*. The mean of 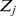 for all markers in bin *k* is
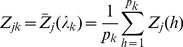
(9)


The total effect of all markers in the bin is
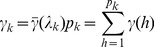
(10)


The working model for *m* bins becomes
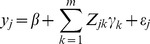
(11)in which all parameters can be estimated provided that 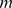 is not too large.

From model (10), we can see that the effect of bin *k* is the sum of effects of all markers within that bin. Two assumptions are required for this infinitesimal model to work: (1) high linkage disequilibrium and (2) homogeneous effects of markers within a bin. For example, if the effects of individual markers are not in the same direction, positive and negative effects may be cancelled out each other, leading to a zero net effect for the bin. The first condition (high linkage disequilibrium) is satisfied in line crossing data analysis (QTL mapping), especially in F_2_, BC and DH populations. Recombinant inbred lines (RIL) usually have low linkage disequilibrium and thus application of the infinitesimal model to RIL is problematic. The second condition (homogeneous effect) appears to be out of our control. However, we can choose bin sizes as small as a program can manage so that the chance of more than one markers having effects per bin is minimum. This will at least avoid cancellation of effects in opposite directions. If the number of QTL in the entire genome is not extremely large and the locations of these QTL are randomly distributed along the genome, a small bin containing more than two QTL may have a negligible probability, and thus the second condition may well be satisfied. Theoretical investigation of the infinitesimal model is provided in the next section. Although 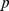 can be infinitely large, the reduced model has a dimensionality of 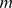. A model with a small number of effects can be handled by the ordinary least squares method. If 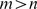, a penalized regression may be used, e.g., the Lasso method [16] or the Bayesian shrinkage method [12]. The idea of the proposed bin analysis is to reduce the model dimension from a virtually unlimited number of markers to a finite number of bins that can be managed easily using existing software packages.

### Theory of the Infinitesimal Model

We now show the theoretical basis of the infinitesimal model and explain why this model requires the two assumptions (high linkage disequilibrium and homogenous effects within a bin). We now use an F_2_ population derived from the cross of two inbred lines as an example to demonstrate the theory. The genotype indicator variable for marker *h* within bin *k* is defined as
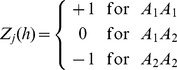
(12)


The average of the indicator variables for all the 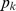 markers within bin *k* is
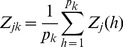
(13)


The variance of 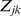 across individuals determines the efficiency of the model. Let 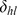 be the recombination fraction between loci *h* and *l* within bin *k*. The variance of 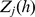 is 1/2 and the covariance between 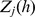 and 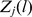 is 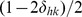. The variance of 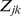 across individuals is
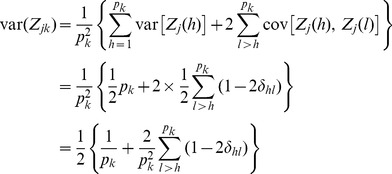
(14)


This variance will take a value between 0.5 and 0 corresponding to 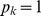 and 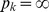, respectively. When 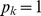, the second term of the above equation will vanish and thus 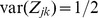. The bin analysis becomes the individual marker analysis. When 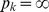 and the 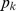 markers do not overlap, then 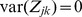. However, the reduction of the variance also depends on the pair-wise recombination fractions. If all the markers within the bin are jammed together (complete linkage), we have
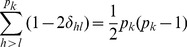
(15)which also leads to 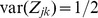. It is well known that the statistical power of a simple regression analysis depends on the variance of the independent variable. A larger variance of the independent variable leads to a higher power to detect the regression coefficient. In the proposed bin analysis, the independent variable is 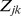 and thus a larger 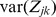 corresponds to a higher power for bin detection. The high linkage disequilibrium of markers within a bin will slow down the reduction of 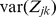 from 1/2 to 0. This is why a high linkage disequilibrium within a bin is required for the infinitesimal model.

We now discuss why homogeneous effects of markers within a bin are required. Recall that we defined
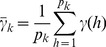
(16)as the average effect of all markers within bin *k*. The bin effect is 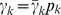, the total effects of all markers within that bin. When the marker effects within the bin are heterogeneous, especially when they are in opposite directions, the average value may end up being zero. Therefore, homogeneous marker effects within a bin are important; at least, they should not be in opposite directions. The exact genetic value of bin *k* for individual *j* is
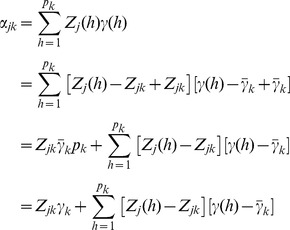
(17)where 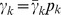 is the total genetic effect for bin k. The second term in the above equation is the sum of cross products of 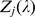 and 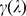 in the bin, denoted by 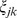




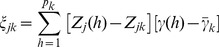
(18)The bin implemented infinitesimal model actually assumes 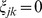 and thus 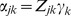. Either one of the two assumption described early will make the ignorance safe. Under the assumption of high linkage disequilibrium, 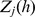 is pretty much a constant across the markers within the bin (not a function of *h*) and thus
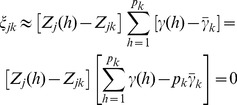
(19)


Under the assumption of homogeneous marker effects within a bin, the second condition, 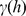 is pretty much the same across all markers within the bin. Therefore,
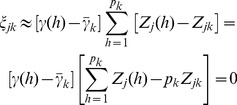
(20)


The above discussion seems to be contradictory to the main purpose of the proposed bin analysis. The maximum power is obtained by choosing bins that contain only a single marker per bin. This would end up with an infinite number of bins and thus the model is not a working model. What we want here is to decide a finite number of bins that can be handled by a program, yet the power should not be lost too much: a new way to handle an infinite number of markers.

### Concept of the Adaptive Infinitesimal Model

We now modify the infinitesimal model so that it can analyze infinite number of markers for populations in low or no linkage disequilibrium. Let us define a weighted average of 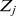 for all markers in bin *k* by
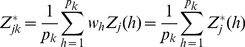
(21)


The weighted average effect for this bin is defined as
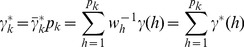
(22)where 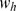 is a weight assigned to marker *h* within bin *k*. The working model under the weighted strategy is
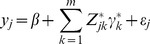
(23)Since 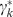 is the effect of bin k and it is a quantity subject to estimation, we really do not need 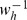. We only need 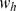 to find the weighted average of the genotype indicator variable 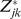. As a result, the weight can take zero as a legal value. The weighted model actually estimates the sum of the weighted effects of all markers within a bin. Our purpose here is to choose a weight so that the effects within the bin are homogenized. The weight should also be chosen from a preliminary analysis of the same data. With a proper selection of the weight, the two assumptions described in the unweighted infinitesimal model may be relaxed. There might be many different ways to choose the weight, but we proposed the following weight,
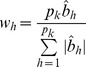
(24)where 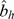 is the least squares estimate of the hth marker in a single marker analysis. A special property of the weight is 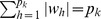. Because the weights depend on the least squares estimates of the marker effects and thus depend on the data, we call the modified model the adaptive infinitesimal model. The theory behind the adaptive infinitesimal model is given below.

### Theory of the Adaptive Infinitesimal Model

We now show that the adaptive model can homogenize the marker effects within a bin so that it can handle populations in low or no linkage disequilibrium. Let us redefine the effect of bin *k* for individual *j* by
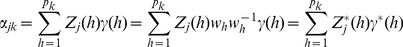
(25)where 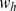 is a weight for marker *h*, 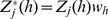 is a weighted genotype indicator variable and 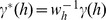 is a weighted effect. Let us define
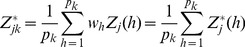
(26)as the weighted average indicator variable for bin k and
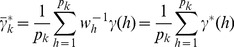
(27)as the weighted average of all marker effects in bin k. The total effect of bin k is 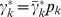. Let us rewrite equation (25) as
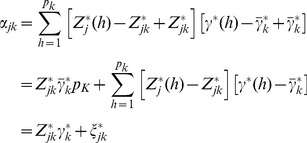
(28)where



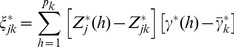
(29)Recall that the weight is,
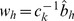
(30)where 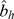 is the least squares estimate of the effect of the *h* ^th^ marker in a single marker analysis and
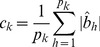
(31)is the average of the absolute values of the least squares estimates of all marker effects within bin k. This weight leads to 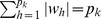. The weighted effect for marker h in bin k is



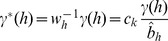
(32)If the least squares estimate 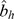 is proportional to 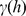, the ratio 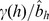 will be roughly a constant across all markers within the bin, i.e., 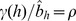 for all 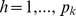. This is what we called homogenization of the effects. The homogenization will lead to
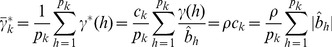
(33)


Therefore, the bin effect defined by 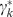 is
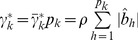
(34)which is roughly proportional to the sum of the absolute values of all markers in bin *k*. In addition, we can safely assume 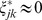 because of the homogenization. The adaptive working model with *m* bins is now expressed as



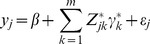
(35)Because 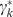 is either zero or positive, it is called the score of bin *k*, which can be tested using any regression methods that can handle 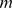 bin effects.

### Parameter Estimation and Cross Validation

When the number of bins is much smaller than the sample size, the effects of the bins can be estimated using the ordinary least squares method. However, the number of bins should be sufficiently large to achieve a high resolution for the purpose of QTL mapping. Therefore, the situation is often reversed in reality, i.e., the number of bins is often larger than the sample size. For example, if we choose a bin size of 1 cM, the entire human genome will be divided into over 3000 bins. To estimate 3000 effects in a single model using the ordinary least squares method, we need at least 3000 subjects just to make sure exist of a unique solution of 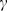. We may need a sample size of at least 5000 to get a reasonable good estimate of 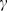. If the number of bins is larger than the sample size, we need a penalized regression method, e.g., the Lasso method [16] and the Bayesian shrinkage method [12]. In this study, we chose the Lasso method implemented in the GLMNET/R package [17] for parameter estimation because of its fast computational speed.

Although the main purpose of the infinitesimal model is to predict the genomic value, the method can serve as a QTL mapping procedure. A QTL effect in a traditional mapping experiment is simply replaced by a bin effect in the current model. The GLMNET/R program does not provide such a test statistic and thus we calculated the test statistics from the output of the GLMNET/R program. We first calculated the estimation error for each bin effect using 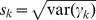, where the variance is approximated by
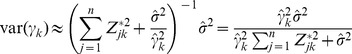
(36)and 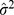 is the estimated residual error variance. The Lasso estimate often generate many zero estimated bin effects, for which the estimation errors are forced to be zero and thus tests are not performed for those zero effect bins. Given the estimated effect 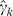 and its error 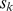, we are able to calculate the t-test statistic or its square, the F-test statistic. The F-test statistics is equivalent to the Wald test [18] when the numerator degree of freedom is one. Therefore, we can present the test statistic as the Wald test statistic,



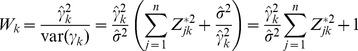
(37)This test is also very much like a likelihood ratio test statistic and thus it can be transformed into a LOD score test
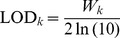
(38)


As a convention in LOD score test, we may choose LOD  = 3 as the criterion to declare statistical significance for each bin.

For purpose of genomic prediction, we used the mean squared error (MSE) obtained from the 10-fold cross validation [19] as a criterion to evaluate the predictabilities of the models under various bin sizes. In the first step of the cross validation, individuals of the population were random partitioned into 10 subsamples (parts). In the second step, we used nine parts of the sample to estimate parameters and used these estimated parameters to predict the phenotypes of individuals in the remaining part. The cross validation concluded after all parts have been predicted. The MSE [20] is defined as
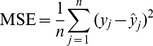
(39)where
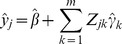
(40)is the predicted value of individual j using parameters, 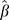 and 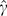, that are estimated from samples excluding individual j. A small MSE means a better prediction. The MSE can be transformed into a quantity between 0 and 1, with 0 being no predictability and 1 being prefect prediction. This quantity is denoted by 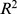 and expressed by
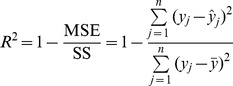
(41)which can be interpreted as the proportion of the phenotypic variance contributed by all the bins and thus markers of the entire genome.

### Competing Methods of Genomic Selection

The adaptive infinitesimal model was compared to five competing models commonly used in genomic selection. The five competing models are: (1) empirical Bayes (eBayes) [21], (2) ridge regression [22], also called BayesA [11] or G-Blup [23], [24], (3) BayesB-1 [11], (4) BayesB-2 [11] and (5) least absolute shrinkage and selection operator (Lasso) [16], [17]. For the paper to be self-contained, we briefly describe these competing methods in this section. The eBayes method is a mixed model approach by treating intercept and co-variates as fixed effects and the marker effects as random effects with independent marker specific normal distributions, i.e., each marker effect has a normal distribution with mean zero and its own variance. It is called empirical Bayes because the marker effects are considered as parameters and their normal distributions considered as prior distributions. The method estimates the variance components first, independent of the marker effects, and then uses the estimated variance components as prior variances to generate Bayesian estimates of the marker effects. The G-Blup method is essentially the same as the eBayes method except that all markers share the same normal distribution with mean zero and the same variance for all markers. The BayesB methods use a mixture prior distribution for each marker effect (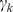),
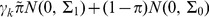
(42)where 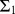 is a variance either estimated from the data or set as a large constant and 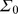 is a small positive number (a constant close to zero). The parameter 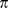 is the proportion of markers with effects large enough to be included in the model. If 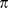 is estimated from the data by using BayesCΠ [25], the BayesB method is called BayesB-1 in this study. If the 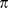 value is set as a constant, say 0.95, the BayesB method is called BayesB-2. The Lasso method can be interpreted as the eBayes method except that the marker specific variance 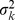 is assigned an exponential prior distribution. The eBayes, the G-Blup and the Lasso methods are rule based methods in the sense that parameter estimation is obtained via an iteration process up to convergence. BayesB-1 and BayesB-2 are stochastic approaches via a Markov chain Monte Carlo (MCMC) sampling process. As a result, the BayesB methods are computationally very intensive.

The eBayes method was implemented via the SAS/IML program of Xu [21]. The Mixed Procedure in SAS (PROC MIXED) [26] was used to perform the G-Blup analysis. The BayesB and BayesCΠ methods were implemented using the online tool GenSel provided by the BIGS project at Iowa State University (http://bigs.ansci.iastate.edu). The 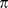 value for the BayesB-1 method was obtained from the data by using BayesCΠ. For the BayesB-2 method, 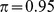 was used, which is suggested by the GenSel investigators as the default value.

## Results

### Simulated Data Analysis

#### Design I: Oligogenic model

**Figure 1:**
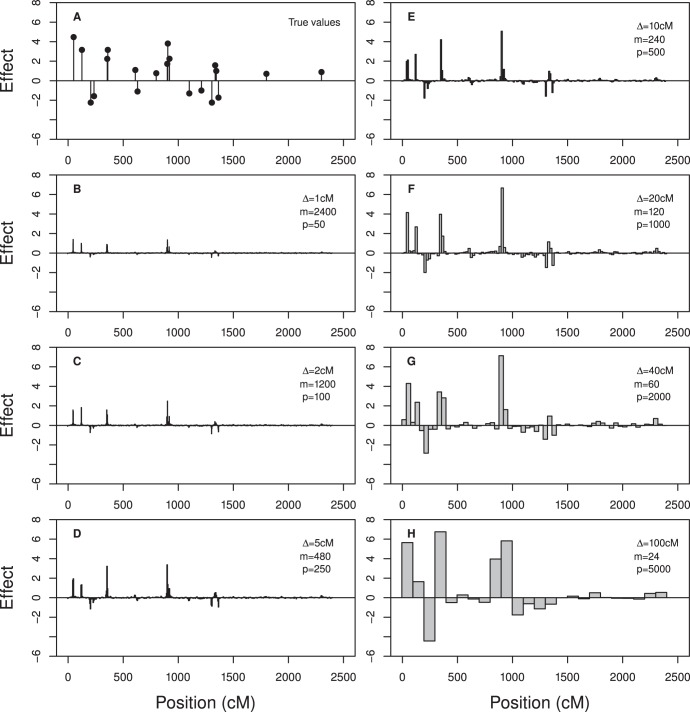
True QTL effects and estimated bin effects for design I. The bin effect estimates were obtained from 100 replicated simulations. The total number of markers was 120,001. The sample size was 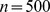. The pure environmental error variance was 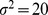. The 20 simulated QTL collectively explained 0.581 of phenotypic variance. The three symbols represent the size of bin (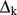), the number of markers (model dimension) within a bin (m) and the number of markers per bin (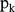). Since the bin size and number of markers per bin are constant across bins, the subscript k has been removed from the figure legends.



In this experiment, we simulated several QTL in the genome with effects varying from small (explaining 1% of phenotypic variance) to large (explaining 15% of phenotypic variance). This experimental setup mimics the usual experimental setup for QTL mapping. In this experimental design, we simulated an F_2_ population (high linkage disequilibrium) with various sample sizes, 200, 300, 400, 500 and 1000. A single large chromosome was simulated with 2400 cM in length. We placed 120,001 SNP markers evenly on the genome with one marker per 0.02 cM. We simulated 20 main effect QTL with positions and effects depicted in Figure 1 (panel a). In addition, we generated 20 pair-wise interaction (epistatic) effects. Collectively, the 20 main effect QTL contributed 64.12 of the genetic variance and the 20 epistatic effects contributed 26.52 of the genetic variance. Since we only estimated the main effects, the epistatic effects would go to the residual (ignored). The proportion of phenotypic variance contributed by the 20 main effects was determined by

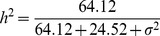
(43)where 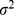 is a pure random environmental error variance. We chose four levels of the 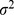 to control 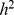. The four levels of 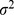 were 10, 20, 40 and 100, corresponding to four levels of 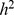, 0.64, 0.58, 0.49 and 0.34. The bin size was chosen at the following seven levels, 1 cM, 2 cM, 5 cM, 10 cM, 20 cM, 40 cM and 100 cM. These bin sizes corresponded to 50, 100, 250, 500, 1000, 2000 and 5000 markers per bin. The corresponding numbers of bins for the seven bin sizes were 2400, 1200, 480, 240, 120, 60 and 24. The largest model contained 2400 bins and the smallest model contained just 24 bins. The GLMNET/R [17] program was used to analyze the data. We chose the Lasso option of the program (L_1_ penalty) using a shrinkage factor (lambda value) of 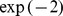 that was predetermined via cross-validation implemented by the GLMNET/R program.

We now present the result of the simulation experiment under sample size 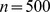 and heritability 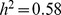 (corresponding to a pure environmental error variance 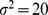). The bin size ranged from 1 cM to 100 cM. The simulation experiment was replicated 100 times and the average estimated effects of the bins across the replicates were represented. The true effects of the 20 QTL are given in Figure 1 (panel a). The estimated bin effects under different bin sizes are also given in Figure 1 (panels b – h). For bin size of 1 cM, corresponding to 2400 bins, the visual plots of the estimated bin effect against the genome location is given in Figure 1 (panel b). Compared with the true effects (panel a), the estimated bin effects showed similar pattern, but with seriously downward biases. Many small to median sized QTL were missed. As the bin size increased, the estimated bin effects visually matched the true QTL effects more closely until the bin size reached about 20 cM (panel f). Further increasing the bin size caused a reduction of the resolution of bin effect estimation. When the bin size reached 100 cM (panel h), some QTL were combined into the same bins and much of the resolution were lost. The estimated bin effects were larger than the simulated QTL effects due to the low resolution. The conclusion was that there appears to be an optimal bin size, at which both the estimated effects and the patterns (locations) of the bins closely match the simulated QTL effects. The close match was obtained visually at the moment. In the next paragraph, we will use the mean squared error (MSE) to evaluate the closeness of match under various bin sizes.

**Figure 2:**
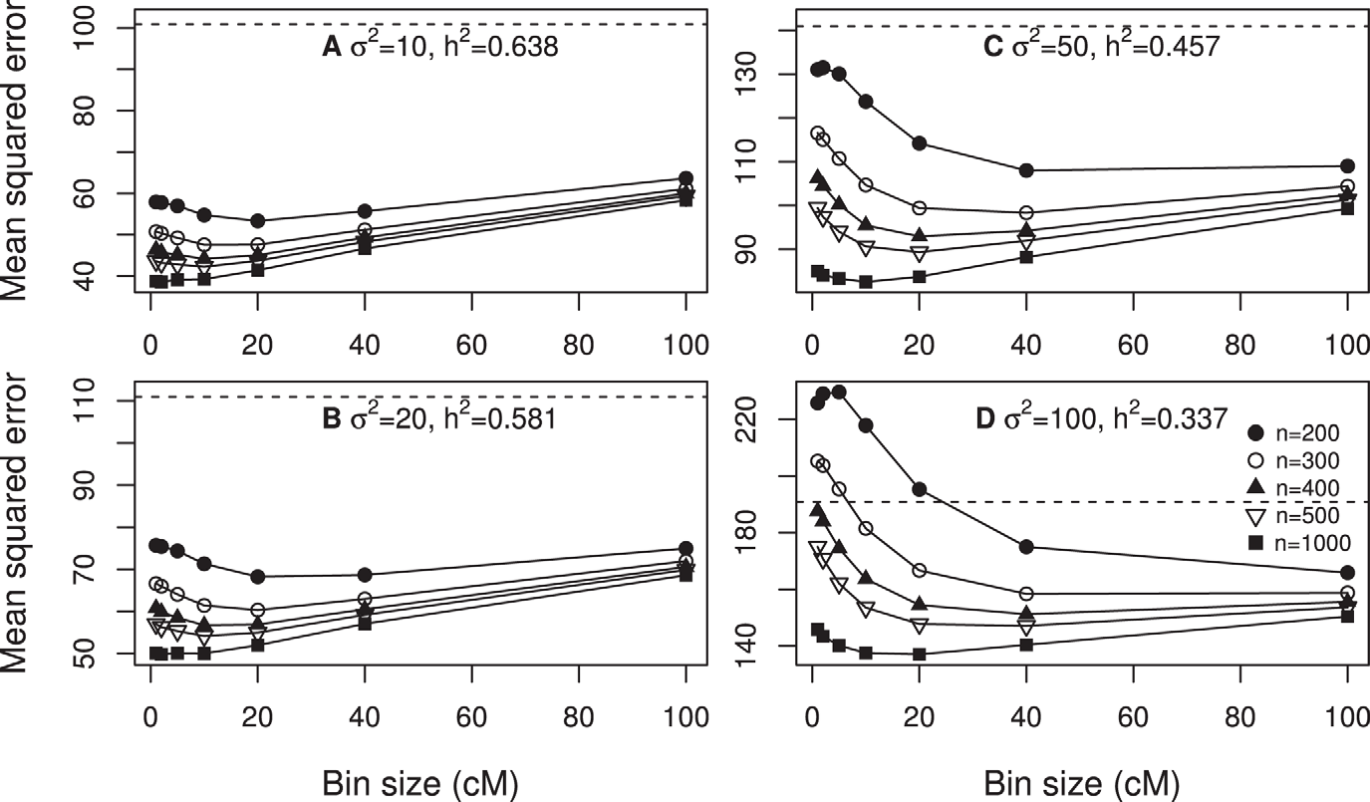
Mean squared error expressed as a function of bin size for design I. The mean squared errors were obtained from 100 replicated simulations. The overall proportion of the phenotypic variance contributed by the 20 simulated QTL was calculated using 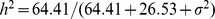. Each panel contains the result of five different sample sizes (*n*). The phenotypic variance of the simulated trait is indicated by the dashed horizontal line in each panel (each panel represents one of the four different scenarios).

The best measurement of the predictability of a model is the MSE. We now examine the effect of bin size on the MSE of the infinitesimal model under various sample sizes and various levels of heritability. Figure 2 shows the MSE under four different levels of the residual error variance (and thus four different levels of heritability). When the residual error variance 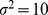 and 20, corresponding to high heritabilities, the MSE curves all have a minimum value at around bin size of 10 cM to 20 cM. When 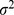 increased to 50 (corresponding to low heritability), the minimum MSE shifted to bin size of 20 cM to 40 cM. When the sample size was 200, the MSE can be larger than the phenotypic variance by chance for smaller bins. Further increasing 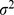 to 100 caused the minimum MSE to shift further to the right. The conclusion from Figure 2 is that the size of bin influences the MSE and the pattern in the change of MSE also depends on the sample size and the heritability.

#### Design II: Clustered polygenic model

**Figure 3:**
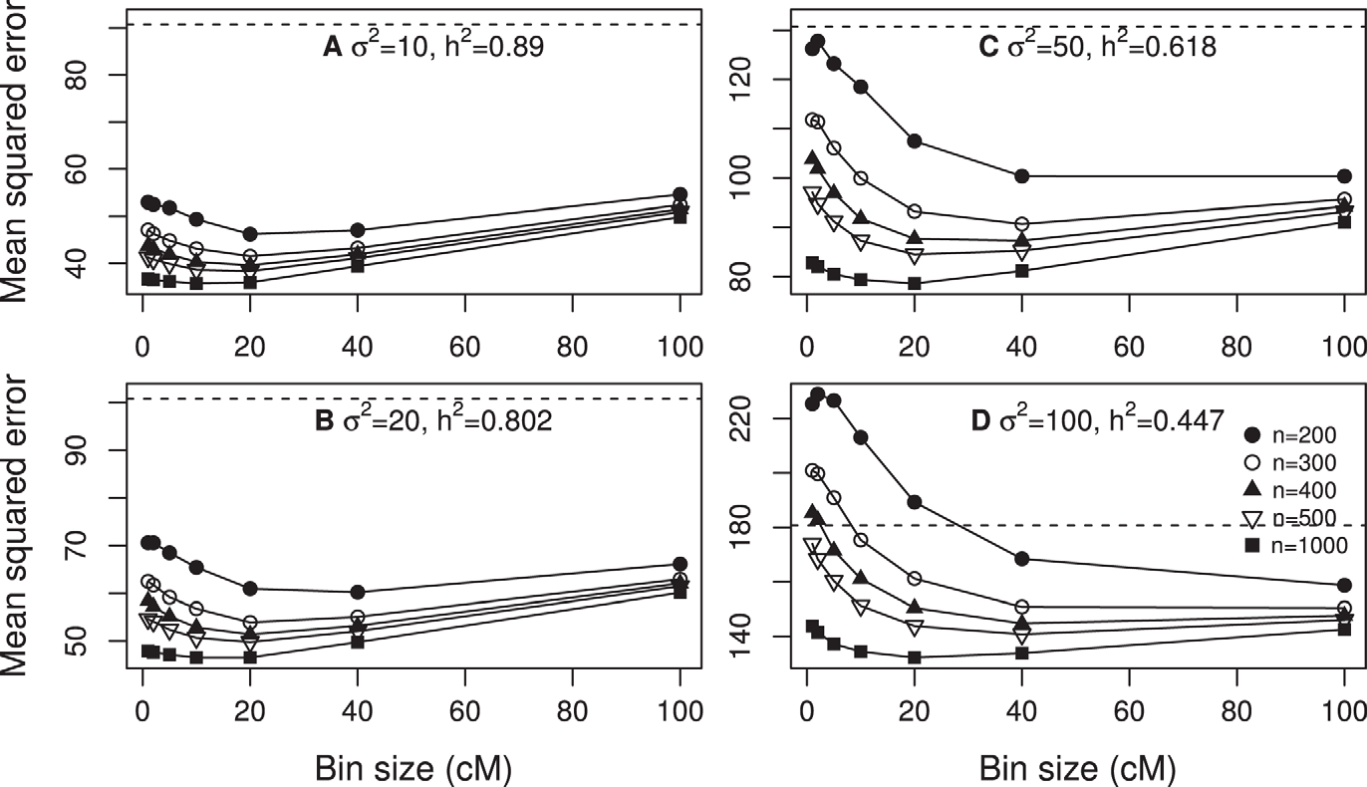
Mean squared error expressed as a function of bin size for design II. The mean squared errors were obtained from 100 replicated simulations. The overall proportion of the phenotypic variance contributed by all simulated QTL was calculated using 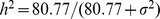. Each panel contains the result of five different sample sizes (*n*). The phenotypic variance of the simulated trait is indicated by the dashed horizontal line in each panel (each panel represents one of the four different scenarios).

In this design, we split each of the 20 QTL given in Design I into 500 equal sized small QTL within ±5 cM of the original QTL position. In other words, each of the 20 QTL in Design I was replaced by a cluster of equal sized small QTL in Design II, which explains why the model is called clustered polygenic model. The total genetic variance contributed by all these small QTL was 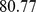. The heritability was calculated using 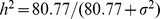, which is a function of the residual error variance. Four levels of 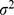 was investigated, including 10, 20, 50 and 100, corresponding to four different levels of 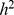, 0.89, 0.80, 0.62 and 0.45. Five different samples sizes were investigated, including 200, 300, 400, 500 and 1000. Figure 3 (panels a, b, c and d) shows the MSE plotted against the bin size. The general observation is that there seemed to be an optimal bin size that produced a minimum MSE. The optimal bin size shifted towards the right as the sample size increased. Exception of this general trend occurred when the sample size was small and the heritability was low (see panel d of Figure 3).

#### Design III: Polygenic model

**Figure 4:**
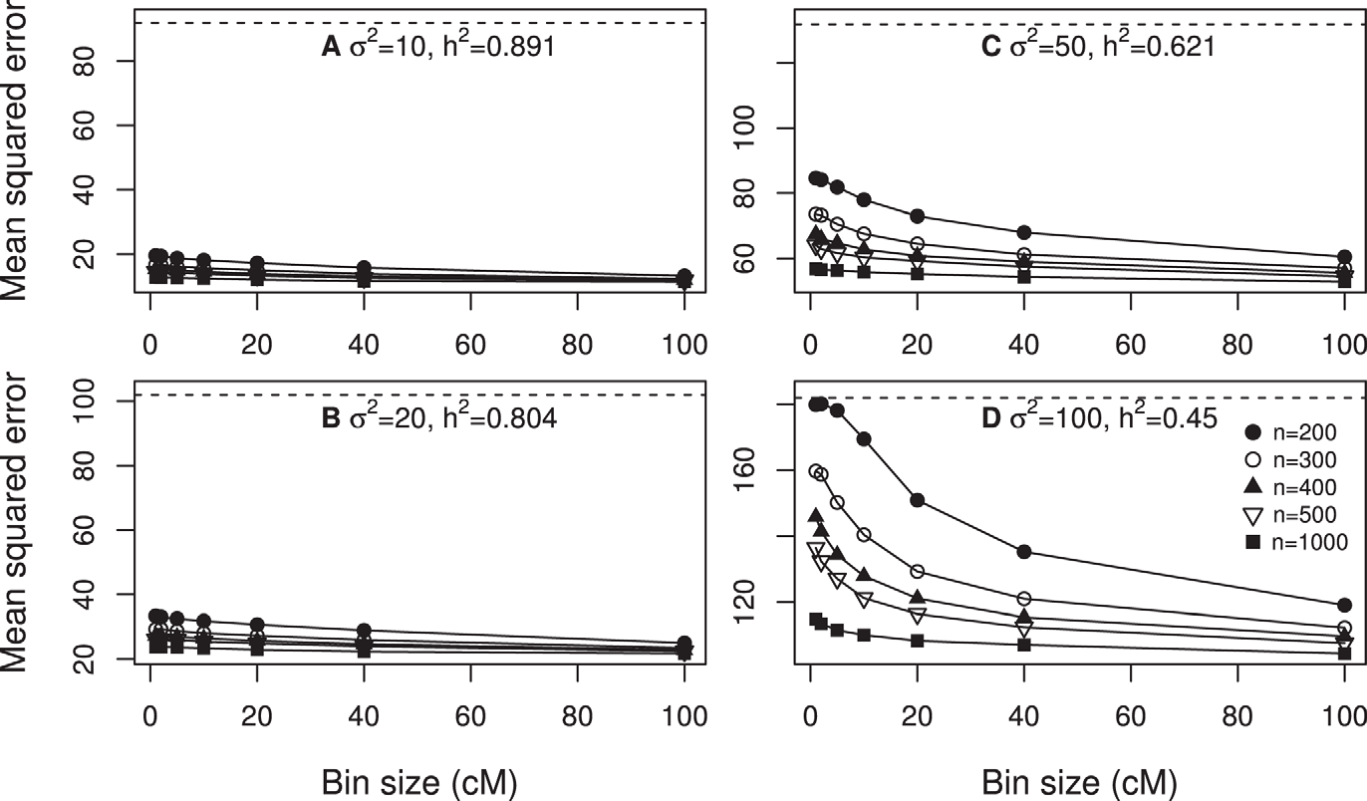
Mean squared error expressed as a function of bin size for design III. The mean squared errors were obtained from 100 replicated simulations. The overall proportion of the phenotypic variance contributed by all simulated QTL was calculated using 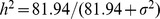. Each panel contains the result of five different sample sizes (*n*). The phenotypic variance of the simulated trait is indicated by the dashed horizontal line in each panel (each panel represents one of the four different scenarios).

Under this design, we simulated 1000 normally distributed QTL evenly placed on the genome. Each QTL explained 1/1000 of the total genetic variance (81.94). The total heritability was calculated using 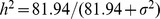. The first 500 QTL had positive effects (located on the first half of the genome) and the second 500 QTL had negative effects (located on the second half of the genome). The heritability is a function of the residual error variance. Four levels of 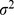 was investigated, including 10, 20, 50 and 100, corresponding to four different levels of 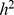, 0.89, 0.80, 0.62 and 0.45. Five different samples sizes were investigated, including 200, 300, 400, 500 and 1000. Figure 4 (panels a, b, c and d) shows the MSE plotted against the bin size. The general observation is that the minimum MSE occurred when the bin size was 100 cM, which was the largest bin in the simulation experiment. When the heritability was high, the changes of MSE did not seem to be large across different sizes of bin. Further increasing the bin size actually increased the MSE (data not shown).

#### Design IV: Oligogenic model under low linkage disequilibrium

**Figure 5:**
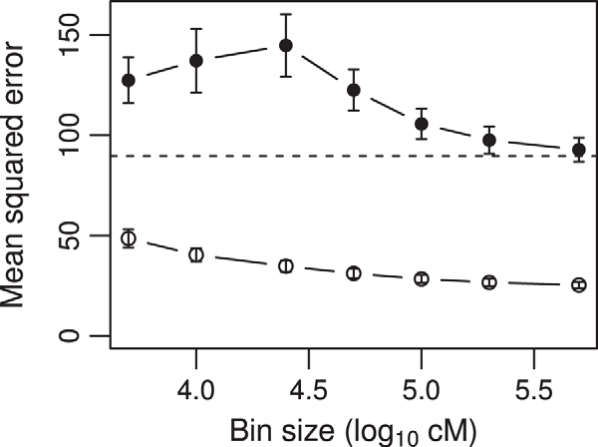
Mean squared error for the simulated data under design IV plotted against the bin size. Design IV was for populations with low levels of linkage disequilibrium. The sample size of the simulated population was 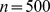. The residual error variance was 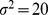, corresponding to 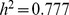. The filled circles indicate the MSE under the infinitesimal model while the open circles indicate the MSE under the adaptive infinitesimal model. The dashed horizontal line represents the phenotypic variance of the simulated trait (89.71).

This design was similar to Design I except that we now expanded the genome from 2400 cM to 12,000,000 cM, which was 5000 times the genome size of Design I. In addition, we did not simulate epistatic effects in this design. The number of markers remained 120,001 but each marker interval covered 100 cM. The linkage disequilibrium level was very low or null. We expected that the infinitesimal model would break down due to the low linkage disequilibrium, but the adaptive infinitesimal would fix it. We simulated 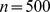 individuals under a residual error variance of 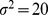, corresponding to 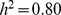 for all the 20 simulated QTL. The experiment was replicated 100 times. This time, we only presented the MSE to show the difference in predictability of the infinitesimal model and the adaptive infinitesimal model. Figure 5 shows the plot of the MSE against the bin size (log_10_ cM) for both the infinitesimal model (filled circles) and the adaptive infinitesimal model (open circles). The infinitesimal model had all MSE above the actual phenotypic variance (89.71, indicated by the horizontal line in the middle of the figure). When the MSE is larger or equal to the phenotypic variance, it means no predictability. This simulation study did show the failure of the infinitesimal model under low linkage disequilibrium. We now evaluate the adaptive model under this low linkage disequilibrium design. The open circles represent the MSE under the adaptive infinitesimal model. All the MSE were smaller than the phenotypic variance, indicating that the adaptive model was effective for low linkage disequilibrium. The MSE actually decreased as the bin size increased. For the smallest bin (largest number of bins), the MSE was 48.53, corresponding to an 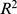 value of 0.459. As the bin size increased (number of bins decreased), the MSE progressively decreased until it reached 25.31, corresponding to an 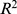 value of 0.718. The predictability was consistently improved as the bin size increased. However, we expect that further increasing the bin size would decrease the predictability.

### Carcass Weight Analysis in Beef Cattle

The carcass weight data used in this study were resulted from residual feed intake (RFI) trials on 922 beef composite steers at the Kinsella Research Ranch of the University of Alberta, Canada from 2004 to 2009. These beef composite steers were progenies from crosses between Angus, Charolais, or University of Alberta hybrid bulls and the University of Alberta’s experimental hybrid dam line. The phenotypic observations of carcass weight were collected in the abattoir, which was described by Nkrumah *et al*. [27], [28], [29]. DNA samples were extracted from blood samples of the 922 beef steers during the feedlot trials. High-throughput genotyping was carried out at the Bovine Genomic Laboratory at the University of Alberta using the Illumina BovineSNP50 BeadChip. Those SNP that were not mapped to the Btau4.0 reference assembly (http://www.hgsc.bcm.tmc.edu/ftp-archive/Btaurus/fasta/Btau20070913-freeze/) were excluded from the analysis. All SNP with minor allele frequency (MAF) <0.05 were removed from this analysis [30]. After the filtering with the above criterion, a total of 40,809 SNP remained for the analysis. A total of 86 animals with missing phenotypic observations were excluded from the analysis, resulting in 836 animals with carcass weight observations subject to the analysis. Co-factors considered in the analysis included sire breed, test group and the slaughter age. The systematic effects of the three co-factors were pre-adjusted via a linear model analysis and the residuals resulted from the linear model adjustment were subjected to the analysis.

**Figure 6:**
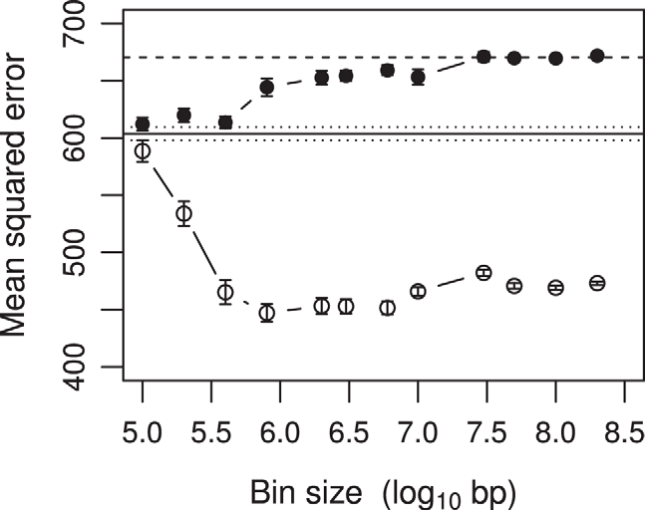
Mean squared error for the carcass trait of beef cattle plotted against the bin size. The filled circles indicate the MSE under the infinitesimal model while the open circles indicate the MSE under the adaptive infinitesimal model. The dashed horizontal line represents the phenotypic variance of the simulated trait (670.36). The solid horizontal line along with the two dotted lines represents the MSE and the standard deviation of the MSE in the situation where the bin size was one (one marker per bin). The sample size was 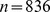 and the number of SNP markers was 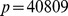. The bin size was defined as log_10_ bp. For example, the largest bin size 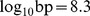 means that the bin size contains 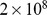 base pairs.

The sample size was 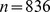 and the number of SNP markers was 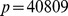. The sample size was sufficiently large to handle all the 40809 markers without using the bin analysis by Lasso method implemented in the GLMNET/R program. However, we still performed the bin analysis by defining various bin sizes with a 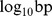 unit. Phenotypic variance of the carcass trait after adjusting for the fixed effects was 670.36. The MSE under both the infinitesimal model (filled circles) and the adaptive infinitesimal model (open circles) are presented in Figure 6. First, all the MSE values were below the phenotypic variance (670.36, dashed line), meaning that both models were useful for prediction. Secondly, the MSE under the marker analysis (one marker per bin) was about 603.75 (the blue horizontal line), less than the phenotypic variance (the dashed line). The corresponding 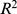 value was 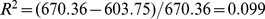, meaning that the 40809 markers collectively explained only about 9.9% of the phenotypic variance for the original marker analysis. Thirdly, as the bin size increased, the MSE of the infinitesimal model (filled circle) progressively increased until it reached the phenotypic variance (670.36), e.g., no predictability. This analysis showed that the beef population had some level of linkage disequilibrium, but not high enough to make the unweighted bin analysis more effective. Finally, we evaluated the MSE of the adaptive infinitesimal model. In contrast to the infinitesimal model, the adaptive model showed a sharp decrease in MSE as the bin size increased. The MSE reached the minimum value of 447.10 when the bin size was about 5.9 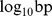. The corresponding R-square was 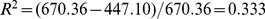, meaning that the bin effects collectively explained 33.3% of the phenotypic variance. As the bin size further increased, the MSE progressively increased. The bin analysis showed a significant improvement of the predictability of the model. The results of MSE and R-squares were obtained from the multiple regression models in the sense that all model effects were included in a single model. The software package used is GLMNET/R [17].

**Figure 7:**
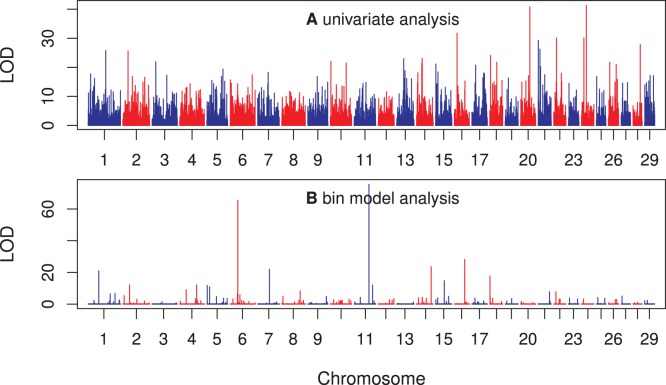
LOD scores of individual markers and bins of the carcass trait of beef cattle. (a) The top panel shows the LOD scores of individual marker analysis (simple regression analysis for each marker). (b) The panel at the bottom shows the LOD scores of the bins obtained from the adaptive infinitesimal model analysis with a bin size of 5.9 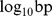 (the optimal bin size). The number of bins under this optimal size was 3186.

Finally, we showed the LOD scores of 3186 bins across the entire beef cattle genome in Figure 7 (panel b). The LOD scores were obtained from the adaptive infinitesimal model analysis with a bin size of 5.9 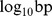 (the optimal bin size). The top panel of Figure 7 gives the LOD scores for all the 40809 markers (not the bins) using a simple regression analysis (not from the Lasso method). The LOD scores of the two analyses do not agree with each other. The individual marker analysis showed that chromosomes 20 and 24 each had a major marker with a very high LOD score. However, the bin model analysis did not show any evidence of major effects for these two chromosomes; instead, chromosomes 6 and 11, each had a major bin with LOD scores over 60. Note that the 40809 estimated marker effects (regression coefficients) corresponding to the LOD scores in this figure were only used to find the weights for the adaptive infinitesimal model. They were not used in the MSE and R-square comparisons.

The infinitesimal model developed here is not a new statistical method. It generates new data (the bin data) and uses the new data to perform genomic selection. Any multiple regression methods can be used to perform the bin model analysis, as long as the model can handle the finite number of bins. We choose the Lasso method because it is computationally more efficient than all other competing methods. We have shown that the bin model is significantly better than the Lasso method that uses the original marker data. One reviewer stated that Lasso is not the standard method for genomic selection and other competing methods should be compared. Both reviewers suggested comparison be made between the bin model and other competing models. Following their suggestion, we compared our bin data analysis with the following competing methods, (1) eBayes, (2) G-Blup, (3) BayesB-1, (4) BayesB-2 and (5) Lasso. Brief descriptions of these methods are given in the methodology section.

Again, we used the MSE and the R-square values generated from the 10-fold cross validation analysis as criteria for the comparison. In all the five competing methods, the original 40809 SNP markers were included in the model. The MSE and the R-square values of all six models (five competing models plus the bin model) are given in Table 1. The MSE of all models are smaller than the phenotypic variance (670.36), meaning that all methods are effective to a certain degree. The bin model has the smallest MSE (447.10), followed by Lasso (603.75), G-Blup (632.46), eBayes (648.11), BayesB-1 (655.59) and BayesB-2 (658.19). The best competing model is Lasso. On contrary to the common belief that BayesB is the best method for genomic selection, this study shows that BayesB is the worst one among these competing models. One may argue that the comparison is not fair because the bin model estimates bin effects rather than marker effects. If we use eBayes and BayesB methods for the bin data, they can be more efficient than the corresponding methods using the marker data. This is exactly the point we are trying to address: the bin model uses new data (bin data) and such a model has not been available yet before this study.

**Table 1:** Mean squared error (MSE) and R-square values obtained from the 10-fold cross validation analysis for the beef carcass trait using five competing models and the proposed bin model.

Model	MSE^2^	R-square
eBayes	648.11	0.0332
G-Blup	632.46	0.0565
BayesB-1	655.59	0.0220
BayesB-2^1^	658.19	0.0182
Lasso	603.75	0.0994
Bin model	447.10	0.3330

1 The Pi value for BayesB-2 is set at 0.95.

2 The phenotypic variance of the beef carcass trait is 670.36. The magnitude of MSE value smaller than 670.36 indicates the effectiveness of the model predictability.

## Discussion

Our analysis shows two bins with very large LOD scores. One bin is on chromosome 6 (bin 900, LOD score 65.48) and the other on chromosome 11 (bin 1652, LOD score 75.54). The first bin covers 19 SNP and the second bin covers 8 SNP. Information of the two bins and the SNP covered by the two bins are presented in Tables S1 and S2. Nine out of the 19 SNP covered by the bin on chromosome 6 have LOD scores >3.0, based on individual marker analyses. Some of the individual effects are positive while others are negative. None of the 8 SNP covered by the bin on chromosome 11 have LOD score >3.0, based on individual marker analyses. Without the bin analysis, none of the 8 SNP in the bin on chromosome 11 would be detected. However, the collective effect of the 8 SNP is very significant. This observation clearly demonstrates the power of the bin model.

We used the Lasso method to analyze the bin data. The other competing methods, except the G-BLUP method, can also be used to analyze the bin data. We anticipated that those methods would also improve the predictability if applied to the bin data compared to the analyses with the original SNP marker data. However, we do not expect these methods to be better than the Lasso method for the bin data based on the results of the SNP marker analysis. It will be an excellent topic for further investigation to compare all available genomic selection procedures under the bin model.

Prior to the genome era, quantitative genetics was dominated by the infinitesimal model [31]. Effects of individual genes were not recognizable and thus the collective effects of genes had to be studied using pedigree information. Genetically related individuals share a proportion of their genetic material and the shared proportion varies as the degrees of relationship varies, which provides the foundation for genetic parameter estimation and breeding value prediction. The best linear unbiased prediction (BLUP) technique [32] marked the peak of the classical quantitative genetics. By the end of 1980’s, the classical quantitative genetics reached its end in terms of methodology development, although it is still the basis for plant and animal breeding and the methods are still effective in modern breeding programs. The advent of molecular technology provided an opportunity to revise the model and quantitative genetics faced a transition from classical quantitative genetics to modern quantitative genetics. This transition was mainly due to the landmark work in QTL mapping by Lander and Botstein [1]. The interval mapping procedure proposed by Lander and Botstein [1] was based on the oligogenic model in which a quantitative trait is controlled by a few major genes plus a collection of many genes with small effects. QTL mapping targets these major genes. Using this QTL mapping technology, people have detected many QTL for many quantitative traits [6], [7], [8], [9]. This means that many quantitative traits are indeed guided by the oligogenic model. For the last two decades, numerous statistical methods have been developed for QTL mapping [33], [34], [35], [36], [37], [38], [39], [40], [41]. These methods mainly addressed the problem of missing genotypes in places where markers are not available (sparse map). With large sample sizes and the high density SNP markers currently available for many species, median and small sized QTL may be detectable now. The problem faced in interval mapping has been reversed; rather than inserting pseudo markers in an interval flanked by two markers, we now have to selectively delete markers because the marker density is too high to be handled by any advanced statistical methods. In the near future, high density SNP data and whole genome sequence data may be available for many species. Advanced statistical methods alone may not be sufficient to deal with the high density markers. The infinitesimal model proposed here serves as a technical preparation to handle such virtually infinite number of markers. Rather than estimating marker effects, we now try to estimate the collective effect of all markers in each interval (bin) of the genome. This requires a new model (the bin model) using currently available statistical methods, e.g., the Lasso method. This new model can take advantage of all markers in the genome and estimate collective effects of all genes regardless how small each gene effect is.

This study emphasizes genomic value prediction. However, the method also applies to QTL mapping. If a trait is indeed controlled by a few large effect QTL, we can detect the bins that contain these QTL (see Design I and Figure 1). Further research can be focused on these significant bins. Each of the significant bins may be further divided into many smaller bins and these small bins are subject to the same analysis. The process may be continued for several iterations until the effects are nailed down to particular markers. For example, the analysis of the carcass trait of beef cattle showed that chromosomes 6 and 11 each had a bin with a large LOD score. In the next step analysis, we may divide each of the two bins into several smaller bins. These smaller bins are then included in a single model for further analysis.

An obvious extension of the model is to investigate epistatic effects by including bin by bin interaction effects. The greatest challenge of epistatic analysis is the high dimension of the model [42]. Since the number of bins can be substantially smaller than the number of markers, including the bin by bin interaction effects can be easily implemented. A bin by bin interaction represents the total epistatic effects of all markers from one bin with all markers from another bin. If the first bin has 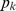 markers and the second bin has 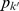 markers, the bin by bin interaction effect is the sum of all the 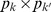 epistatic effects. This requires investigators to calculate the average of 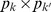 products of genotype indicator variables for the two bins. This step of calculation is performed before the actual data analysis and thus will not significantly increase the computational burden.

The adaptive infinitesimal model was developed for populations with low and no linkage disequilibrium. An obvious question is whether it can also be applied to populations with high linkage disequilibrium. The answer is YES but we do not recommend it. We used the adaptive model to analyze simulated data from Design I, an F_2_ population with high linkage disequilibrium. The results were not as good as the original model analyses in terms of predictability, although the predictability remains relatively high (data not shown). The reason was that the weights introduced for the adaptive model also introduced some noise (estimation errors for the weights). The population disequilibrium level should be lower than a certain level before the benefit appears for the adaptive model. Further study is required to find this level of linkage disequilibrium.

As stated in the main text, there are many different ways to choose the weight for the adaptive model. Consider the estimated effect for marker *h* in bin *k* (denoted by 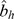), we proposed to use the simple regression analysis for each marker. An alternative method may be the multiple regression analysis for all 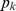 markers simultaneously within each bin. If 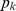 is large, a penalized regression may be used. These alternative methods have not been investigated. Given the 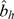
‘s, how to use them to construct the weight 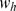 is also worth of further investigation. A simple extension of our weight system may be.
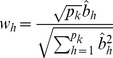
(44)


One property of this weight is 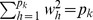. Other properties of this weight system are unknown. A comparison of this weight with our weight will be an interesting project.

Finally, the continuous genome model presented in equation (4) is numerically integrated with a finite number of bins. Within each bin, the number of markers is assumed to be finite also. When the number of markers really approaches to infinity, the data becomes extremely large. Data storage will become a problem, not even mentioning computation. The solution is to use the actual breaking points (recombination events) of the genome as the data. The super saturated marker data will actually tell the breaking points. In fact, it is the actual breaking points that are informative for genetic analysis. Breaking point mapping is a new concept derived from the infinitesimal model. The idea of bin analysis [43], [44] and breaking point mapping will open a new avenue to study unlimited volume of genomic data.

## Supporting Information

Table S1 **Information of two large effect bins on chromosomes 6 and 11.**
(DOC)

Table S2 **Information of SNP covered by the two large effect bins on chromosomes 6 and 11.**
(DOC)
