# Supplementary material for: An Infinitesimal Model for Quantitative Trait Genomic Value Prediction
Source: PLoS One. 2012 Jul 18;7(7):e41336. doi: 10.1371/journal.pone.0041336 (PMC3399838; doi:10.1371/journal.pone.0041336)
Supplement: Table S1 — Information of two large effect bins on chromosomes 6 and 11. (DOC) [file pone.0041336.s001.doc]

**Table S1:** Information of two large effect bins on chromosomes 6 and 11.

| Bin ID | Chr | Position range (bp) | Effect | LOD | Number of SNP |
| --- | --- | --- | --- | --- | --- |
| 900 | 6 | 38533538-39223437 | 11.51 | 65.48 | 19 |
| 1652 | 11 | 73913796-74537774 | 10.58 | 75.54 | 8 |
