# Supplementary material for: An Infinitesimal Model for Quantitative Trait Genomic Value Prediction
Source: PLoS One. 2012 Jul 18;7(7):e41336. doi: 10.1371/journal.pone.0041336 (PMC3399838; doi:10.1371/journal.pone.0041336)
Supplement: Table S2 — Information of SNP covered by the two large effect bins on chromosomes 6 and 11. (DOC) [file pone.0041336.s002.doc]

**Table S2.** Information of SNP covered by the two large effect bins on chromosomes 6 and 11.

| Chr | SNP ID | SNP name | Allele1 | Allele2 | Effect | LOD | Position (bp) |
| --- | --- | --- | --- | --- | --- | --- | --- |
| 6 | 11247 | Hapmap23117-BTC-034857 | A | C | 1.9419 | 0.9621 | 38533538 |
| 6 | 11248 | Hapmap28546-BTC-072715 | G | C | 1.5888 | 0.7719 | 38558526 |
| 6 | 11249 | Hapmap53940-rs29026121 | T | C | 2.0522 | 0.8995 | 38600494 |
| 6 | 11250 | Hapmap27537-BTC-060891 | T | C | 4.6872 | 4.8309 | 38638962 |
| 6 | 11251 | BTB-01709638 | A | G | 4.2845 | 2.5229 | 38658980 |
| 6 | 11252 | ARS-BFGL-NGS-27686 | T | C | -7.9523 | 14.3669 | 38697373 |
| 6 | 11253 | Hapmap31044-BTC-071337 | T | C | 2.8333 | 1.9944 | 38729866 |
| 6 | 11254 | Hapmap33170-BTC-071249 | A | G | 5.1823 | 3.6103 | 38756335 |
| 6 | 11255 | BTB-01326707 | T | C | -7.5976 | 9.9181 | 38824038 |
| 6 | 11256 | Hapmap27849-BTC-071108 | T | C | -6.8365 | 9.5657 | 38888186 |
| 6 | 11257 | Hapmap33339-BTC-071052 | A | G | 4.2553 | 3.4203 | 38914556 |
| 6 | 11258 | Hapmap26618-BTC-070864 | A | G | -4.3367 | 2.6934 | 38982338 |
| 6 | 11259 | Hapmap27344-BTC-063259 | T | C | -5.9928 | 5.5177 | 39052608 |
| 6 | 11260 | BFGL-NGS-113801 | A | G | 1.1321 | 0.3954 | 39072637 |
| 6 | 11261 | Hapmap23923-BTC-066021 | T | G | 1.5738 | 0.5531 | 39108078 |
| 6 | 11262 | Hapmap32513-BTC-066089 | T | C | 7.1316 | 10.5803 | 39139502 |
| 6 | 11263 | Hapmap27298-BTC-035654 | T | C | -4.3473 | 2.7811 | 39159587 |
| 6 | 11264 | Hapmap26842-BTC-035606 | A | G | 5.6186 | 6.3537 | 39202496 |
| 6 | 11265 | Hapmap32210-BTC-035534 | A | G | 3.2584 | 1.6870 | 39223437 |
| 11 | 20996 | Hapmap24000-BTA-150203 | T | G | -1.9535 | 0.9699 | 73913796 |
| 11 | 20997 | ARS-BFGL-BAC-11763 | T | C | 1.1686 | 0.4279 | 73936230 |
| 11 | 20998 | ARS-BFGL-NGS-42213 | A | C | 0.8079 | 0.3749 | 73999711 |
| 11 | 20999 | Hapmap42771-BTA-104230 | T | C | 2.3343 | 1.5455 | 74168963 |
| 11 | 21000 | Hapmap50593-BTA-120868 | A | G | -1.9982 | 1.1581 | 74258954 |
| 11 | 21001 | Hapmap25882-BTA-155396 | T | C | 1.3669 | 0.4589 | 74290576 |
| 11 | 21002 | Hapmap57141-rs29027886 | A | G | -0.4620 | 0.2673 | 74405972 |
| 11 | 21003 | ARS-BFGL-NGS-21081 | T | C | -0.4620 | 0.2673 | 74537774 |
